# Genotype by environment interaction for somatic cell score in Holstein cattle of southern Brazil via reaction norms

**DOI:** 10.5713/ajas.20.0031

**Published:** 2020-05-12

**Authors:** Henrique Alberto Mulim, Luis Fernando Batista Pinto, Altair Antônio Valloto, Victor Breno Pedrosa

**Affiliations:** 1 Department of Animal Science, State University of Ponta Grossa, Ponta Grossa, PR, 84030-900, Brazil; 2 Department of Animal Science, Federal University of Bahia, Salvador, BA, 40170-115, Brazil; 3 Paraná Holstein Breeders Association, APCBRH, Curitiba, PR, 81200-404, Brazil

**Keywords:** Dairy Cattle, Environmental Effects, Genetic Evaluation, Mastitis, Somatic Cell Count

## Abstract

**Objective:**

The objective of this study was to evaluate the genetic behavior of a population of Holstein cattle in response to the variation of environmental temperature by analyzing the effects of genotype by environment interaction (GEI) through reaction norms for the somatic cell score (SCS).

**Methods:**

Data was collected for 67,206 primiparous cows from the database of the Paraná Holstein Breeders Association in Brazil, with the aim of evaluating the temperature effect, considered as an environmental variable, distinguished under six gradients, with the variation range found being 17°C to 19.5°C, over the region. A reaction norm model was adopted utilizing the fourth order under the Legendre polynomials, using the mixed models of analysis by the restricted maximum likelihood method by the WOMBAT software. Additionally, the genetic behavior of the 15 most representative bulls was assessed, in response to the changes in the temperature gradient.

**Results:**

A mean score of 2.66 and a heritability variation from 0.17 to 0.23 was found in the regional temperature increase. The correlation between the environmental gradients proved to be higher than 0.80. Distinctive genetic behaviors were observed according to the increase in regional temperature, with an observed increase of up to 0.258 in the breeding values of some animals, as well as a reduction in the breeding of up to 0.793, with occasional reclassifications being observed as the temperature increased.

**Conclusion:**

Non-relevant GEI for SCS were observed in Holstein cattle herds of southern Brazil. Thus, the inclusion of the temperature effect in the model of genetic evaluation of SCS for the southern Brazilian Holstein breed is not required.

## INTRODUCTION

Mastitis is one of the main diseases affecting dairy herds, causing major losses in the milk production chain [1]. It is responsible for most involuntary disposal cases due to inflammation of the mammary gland and losses in animal productivity caused by the disease [2]. The susceptibility of this gland to pathogens causing the disease is largely dependent on physiological, genetic and environmental factors [3]. As such, the search for more genetically resistant animals becomes an interesting point in the dairy production chain [4].

Despite the search for genetically superior animals regarding this trait being a reality, the advances are still small and obtained mainly by correlation with the somatic cell score (SCS) [5]. Although these somatic cells include epithelial cells and white blood cells, the greatest variations between animals (and even in the same animal in test repetitions) are caused by immune cells that migrate to the milk in response to infections by pathogens that cause mastitis [4], characterizing SCS as a strong candidate for the indirect selection for resistance to mastitis because of the high correlation with the trait [6].

Although environmental influences have been observed in the manifestation of the mentioned trait, they are not taken into account in the genetic analysis models applied in the southern Brazil (one of the main milk basis in the country), which can lead to biases in the genetic information and reduce the response to selection [7]. Such a bias in the information may underestimate or overestimate the genetic values in accordance with the environment under analysis, failing to capture the actual genetic variation of animals in environmental changes, assuming their genetic values as constant in affirming a robustness of the trait for all animals. Genetic variation in environmental changes is known as the genotype by environment interaction (GEI) and informs the possibility of performance of a certain genotype with regard to environmental changes [8]. Reaction norms are one of the methodologies that may inform on the existence of these interactions, whether they are relevant or not.

Reaction norms are a powerful and flexible tool for mod eling the effects of the GEI [9]. From their perspective, the genetic behavioral response of the individual can be visualized as a function of the assumed environmental gradient [10]. With this, it is possible to establish which animal genetically responds better according to the environment in which this animal is exposed, evaluating possible reclassifications, in addition to establishing the persistence and genetic variation of the trait in the course of the environmental gradient.

The objectives of this study were therefore: i) verify the existence of a GEI through reaction norm models using random regression for the SCS trait in animals of the Holstein breed according to the regional temperature; ii) estimate the genetic variance and the heritabilities of the SCS according to changes in the temperature gradient; and iii) analyze the genetic performance of the main bulls used in the region regarding the SCS in accordance with changes in the regional temperature gradient.

## MATERIALS AND METHODS

Using the database of official milk recording from the Paraná Holstein Breeders Association, Brazil (APCBRH), data on somatic cell count from 67,206 primiparous females born between 1990 and 2015, daughters of 936 distinct bulls, were recorded. The relationship matrix was composed of 92,637 animals belonging to 398 herds from 88 cities in southern Brazil. The somatic cell count were transformed to SCS to achieve normality and homogeneity of variance, following the formula: SCS = log_2_ (somatic cell count/100,000)+3.

From an a priori survey based on the methodology of Alvares et al [11], the average annual temperatures of the southern Brazil regions were 17°C in the southern portion and 19.5°C in the northern portion of the covered area. Thus, the evaluated regions could be divided into six environmental gradients separated at every 0.5°C of the average annual temperature. In the present study, these divisions were utilized as control variables and the genetic behaviors of the animal population were verified by various changes through the environmental gradients.

The statistical software SAS [12] was used to adjust the raw data and remove possible abnormal information. Beyond the random additive genetic effect, the effects in the reaction norm model adopted in the analysis included the fixed effects of contemporary group and calving age as a covariate (linear and quadratic). The contemporary groups were created considering the interactions of herd-year-season, with four seasons of calving being considered (i.e., December to February, March to May, June to August, and September to November). The data were checked and animals of unknown parentage, progenies of bulls that only pertain to one herd, and contemporary groups containing fewer than three animals were removed.

Among the environmental gradients, herd connectivity was observed through the genetic presence of the bulls in at least three gradients. Since the animals were of unknown parentage, bulls that had been represented in less than three gradients or bulls that featured only once in the herd were removed from dataset. Finally, each environmental gradient contained included a minimum of 1,000 animals for analysis.

A random regression model was adopted to analyze GEI through reaction norms while assuming the residual variance to be heterogeneous in as many as eight classes of variance. Lastly, the fourth class was used because it presented the best fit under the Legendre polynomials. These polynomials were recently selected to promote the improved convergence of data in orthogonal regressions, as seen in the work of Schaeffer [13].

The reaction norms model, via random regression, is de scribed as follows:

$$y_{ij}={IP}_{ij}+{CG}_{ij}+\sum_{m=0}^{M}\beta_{m}\varphi_{m}(T_{ij})+\sum_{m=0}^{M}\alpha_{im}\varphi_{m}(T_{ij})+{ɛ}_{ij}$$

where *y_ij_*, phenotypic record for animal *i*, progeny of sire *j*; *T_ij_*, average temperature; *φ_m_*(*T_ij_*), mth Legendre polynomial order; *CG_ij_*, contemporary group effect; *IP_ij_*, age at first calving as a covariate; *β_m_* average regression coefficient of order m (m = 0,1,2...M); *α_im_* random regression coefficient of order m for additive genetic effect of animal i; *ɛ_ij_*, error associated to variance class. The matrix representations are given as follows:

y=Xb+Zu+e

assuming that:

$$E=\left[\begin{array}{c} y\\ b\\ u \end{array}\right]=\left[\begin{array}{c} Xb\\ 0\\ 0 \end{array}\right]\;\;\;\text{and }\;\;V\left[\begin{array}{c} u\\ e \end{array}\right]=\left[\begin{array}{cc} K_{rn}⊗A & 0\\ 0 & R \end{array}\right]$$

where *y* is the vector of observation; *b* is the vector of the fixed effects attributed to the contemporary group; *u* is the vector of random effects; *e* is the vector of the residual effects; *X* and *Z* are the incidence matrices for the fixed and random effects, respectively; *K_rn_* is the covariance matrix linked to the random effects for the model parameters of the reaction norms; *A* is the additive numerator relationship matrix; and *R* is the residual variance matrix.

The constituents of additive genetic variance and estimates of direct heritability apply to the different environmental gradients according to the following models:

$${h^{2}}_{t}=\frac{{\sigma^{2}}_{g_{a_{t}}}}{{\sigma^{2}}_{g_{a_{t}}}+{\sigma^{2}}_{e_{t}}}$$

being,

$$\sigma_{g_{a_{t}}}^{2}=\sigma_{a}^{2}+\sigma_{b}^{2}\;{\text{t}}^{2}+2\sigma_{a,b}\;\text{t}$$

where h^2^_t_ is the direct heritability for the trait linked to the temperature gradient t; $\sigma_{g_{a_{t}}}^{2}$ is the additive genetic variance ascribed to level t of the temperature gradient; σ^2^_et_ is the estimate of residual variance attributed to level t of the temperature gradient; σ^2^_a_ is the genetic variance estimate of the intercept component; σ^2^_b_ is the slope estimate of the variance components of the reaction norms; σ_a,b_ is the covariance estimate between the genetic effects of the intercept and slope; and t represents the temperature levels. Also, mixed models of analysis using the restricted maximum likelihood method were applied using WOMBAT software [14]. The breeding value was estimated as follows:

$$u_{ij}=\sum_{m=0}\alpha_{im}\emptyset_{m}\;(a_{ij})\;\text{em que }a_{ij}=\left\{a_{ij}\right\}\sim N(0,A{\sigma^{2}}_{g_{a_{t}}})$$

*u_ij_* is the additive genetic value prediction of animal *i* in the environment j; *α_im_* is the random coefficient regression vector of additive genetic effects; ø*_m_* is the i-esim Legendre polynomial associated to mth order; *a_ij_* random effect associated to animal *i* in the environmental level *j*. *A* is the additive numerator relationship matrix. *σ*^2^*_ga_*_*_t_*_ is the additive genetic variance ascribed to level t of the temperature gradient.

For the regression of bulls through the gradients, the ge netic value estimates at the t levels of the environmental gradient in the analysis of genetic behavior were given by the weighted average of the genetic value of the daughters of each bull at each temperature level. A subset of data with information from the bulls present in all environmental gradients was formed to present a more reliable representation of the genetic value in each temperature gradient. For these analyses, 15 sires with the highest number of daughters distributed by southern Brazil were considered. Finally, the GEI was considered statistically significant if the variance of the slope was significantly different from zero by using a one-tailed t-test with a significance level at 0.05, as demonstrated by Cardoso and Tempelman [15].

## RESULTS

The general descriptive mean and the mean by temperature gradient are presented in Table 1 and the genetic, environmental, and phenotypic variances, and heritability for SCS are presented in Table 2. The overall SCS mean (2.66) presented by animals of the Holstein breed in southern Brazil represents about 79,000 cells/mL of somatic cell count. There was a variation between 2.50 to 2.93 in the SCS during the regional temperature increase, which represents about 70,500 cells/mL to 95,000 cells/mL of milk regarding the number of somatic cells. Animals were found with scores equal to 0 in all regions, which corresponds to productions with somatic cell quantities smaller than 12,500 cells/mL of milk. The region with temperatures around 17.5°C were those showing the lowest score in the evaluation, while the region with temperatures around 19.5°C had the highest score in the evaluation.

**Figure 1:**
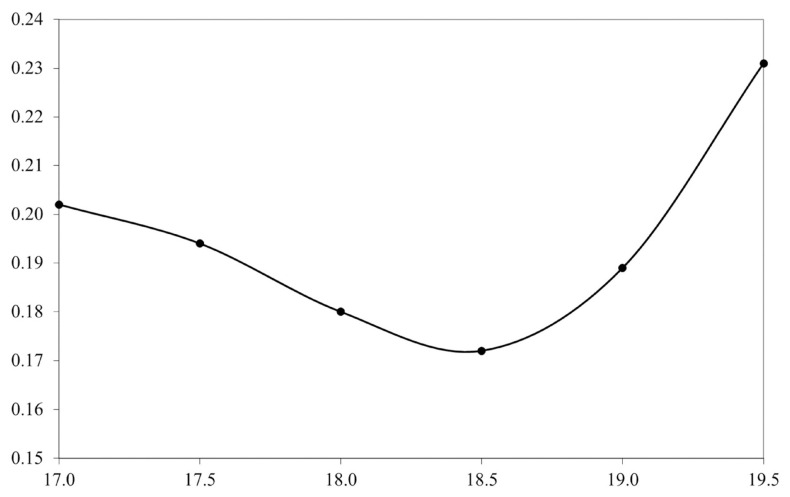
Heritabilities for somatic cell score among the environmental gradients. The x-axis denotes the temperature gradient (°C), and the y-axis represents the heritability estimate. The heritabilities estimates ranges from 0.17 to 0.23 among the temperature gradients.

**Figure 2:**
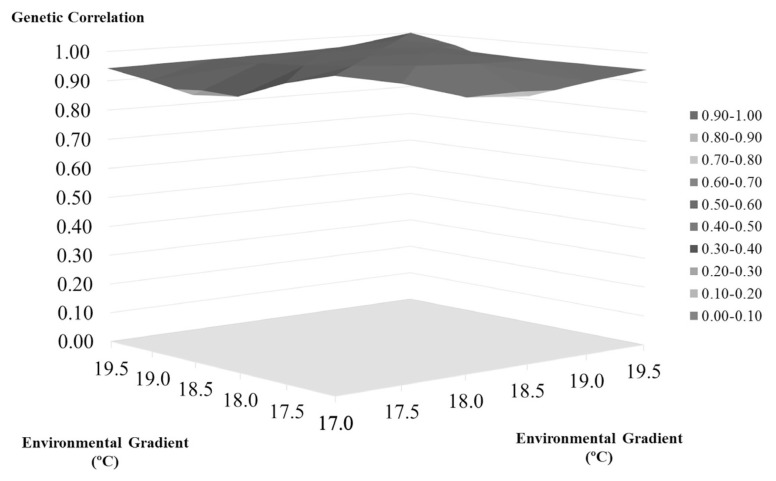
Genetic correlations between the environmental temperature gradients for somatic cell score in the Holstein cattle of southern Brazil. The x-axis denotes the environmental gradient (°C), and the y-axis represents the genetic correlation. The genetic correlation ranges from 0.89 to 0.99 among the environmental gradients.

Table 2 and Figure 1 shows the heritability changes accord ing to the regional temperature increase. As can be seen, the heritability estimates tended to decrease until the 18.5°C gradient with an increase in the heritability estimation occurring after this threshold. The genetic correlation between the environmental gradients used to verify the existence of genotype environment interaction is shown in Figure 2. All the gradients had a correlation above 0.80, with the lowest correlation found being 0.89, between the regions of 17°C and 18.5°C. Correlations close to 1.00 (0.99) were found between the gradients 17.5°C and 18°C and 18.5°C and 19°C. Additionally, based on the t-test for the variance of the slope, no statistically significant G×E was observed for SCS (p>0.05).

**Figure 3:**
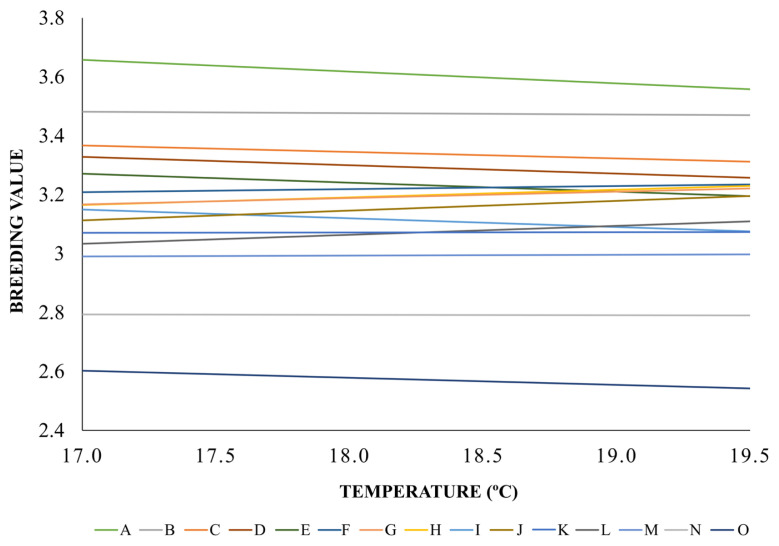
Reaction norms for somatic cell score of the 15 most representative bulls used in southern Brazil region. The x-axis denotes the temperature gradient (°C), and the y-axis represents the breeding value. It was observed an increase of up to 0.258 in the breeding values of the bulls, as well as a reduction in the breeding value of up to 0.793.

The breeding values of the 15 main bulls used in the south ern Brazil and their interaction with the advance of the environmental gradients are presented in Figure 3. It was observed an increase of up to 0.258 in the breeding values of some animals, as well as a reduction in the breeding value of up to 0.793, as well as an increase of up to 0.258, with the changes in the temperature gradient. Most breeding values were close to the score of 3.20, highlighting four bulls with breeding values around this amount in the lower or higher temperature gradient. Bulls A and B had the highest overall breeding values for SCS, while the bulls N and M had the lowest genetic values for SCS as showed in Figure 3.

Lower genetic differences were observed among the animals in the regions close to 19.5°C, demonstrating an approximation of the animal’s breeding values when temperature increases. Changes of specific positions occurred with some animals. Such as in the case of the animal E, which was in fifth position and went to the eighth position along with the bull J, who was in tenth position in the 17°C gradient. The greatest variations in the genetic value of the animals were found for the animals E, F, G, H, I, J, and K, the other bulls had greater stability of their genetic values when the environmental temperature increased.

## DISCUSSION

The means of SCS presented by the Holstein breed in southern Brazil were lower than those established by Brazilian [16] and international legislation [17] for the categorization of type A milk. This indicates that the various producers under study were of high quality, regardless of the region. The score values of “0” identified in all regions highlight the care adopted by the breeders, indicating that values of less than 12,500 cells/mL of milk are found regardless of the temperature under study.

As can be seen, the producers more to the south of the evaluated region (lower temperatures) had the lowest SCS rates. This fact may be related to the existence of traditional dairy basins in these regions [18] with the presence of cooperatives that encourage quality production through bonuses and discounts, which is not so common in the rest of the country. This creates an extra incentive for production with lower SCS rates and higher quality products.

The heritabilities seem to be mostly in the low magni tudes, indicating greater environmental interferences in the manifestation of the trait. Such heritabilities are consistent with recent studies in Brazil [19] and other countries, such as South Korea [20], Iran [21], and Germany [22]. Although these heritabilities are still low, the parameter can be improved not only for SCS, but also indirectly for the resistance to mastitis.

When looking more closely at Figure 1, one can see that the extremities were responsible for the highest heritability rates, different from the common results for the trait in animals of the Holstein breed. This factor may be associated with the use of high-order orthogonal regressions for the analysis of the trait, since these have greater difficulty in estimating values near the extremities of the intervals [23,24]. Meyer [23] mentioned that the tails of the distribution tend to be elevated when compared to the more central information in the distribution. The properties of the polynomials used as those in reaction norms tend to place a large amount of emphasis on the extremes, resulting in inflated estimates. Assuming that the environmental variation sensitivity properties are not constant, we would lose the sensitivity to environmental gradient change properties if the values are regressed linearly on certain environmental gradients, failing to indicate a more accurate representation of the existing variation [25]. Despite this mentioned obstacle, the applied model is indicated for being flexible and able of modeling the changes of the means and variances in a continuous scale [26], better revealing the true behavior in accordance with the change of the environmental gradient.

The correlations, shown in Figure 2, are all above 0.80, indicating the absence of a relevant genotype-environment interaction according to the criteria established by Robertson [27]. This means that the different temperatures evaluated had no effect on the manifestation of the SCS in animals of the Holstein breed in southern Brazil. According to Kolmodin et al [28], it would be hard for dairy cattle factors within a single geographical region to exert effects on the manifestations of characteristics, including on SCS, as we can see here. However, even in the absence of this relevant interaction, it is possible to notice the presence of modifications in the genetic behavior of animals according to the increase in temperature.

As can be seen in Figure 3, distinctive behaviors were observed according to the increase of the environmental gradients. There were bulls who kept their breeding value more constant as the temperature increases, characterizing these animals as more robust regarding the trait [29], which was the case of the animals B, K, M, and N, bulls of greater robustness in the population with nearly zero variations when moving in the environmental gradients. As a counterpoint, the animals with higher variations in the genetic expressions according to the increase of temperature were taken as plastic as they showed modifications to their genetic performances with increases in temperature [30].

In these cases, the animals A, G, H, I, J, and O had the greatest variations in gene expression for the SCS. These variations in gene expression, and even the maintenance of performance according to the increase in temperature, are adaptive responses of the organism to the breeding environment [31] or even of genes that are being expressed in a certain temperature and not in others [30], which affects the genetic behavior of animals over the gradients. The re-ranking occasioned by the changes of the environmental gradients occur depending on the interaction of the animal’s genotype to a particular environment. This is the case of the changes in position of the bulls E, F, G, H, where the temperature had a positive effect on Bull E, decreasing his genetic potential in comparison to the 17°C environment. Different from what was shown for the animals F, G, and H, where the temperature had a negative effect on the genetic performance of the characteristic, increasing the genetic value of animals for SCS.

Despite the small reclassification in the sire ranking and the high genetic correlation observed, first indicating a non-relevant GEI for SCS, a slight change in the heritability estimates thru the gradients were verified. At this moment, the inclusion of the temperature effect in the model of genetic evaluation of SCS is not required, but a constant monitoring of a possible impact of GEI in this trait, for the southern Brazilian Holstein breed, can be recommended.

## Figures and Tables

**Table 1:** Descriptive statistics for somatic cell score in southern Brazilian Holstein cattle

Items	n	Mean	SD	Min	Max
Overall	67,206	2.66	1.48	0.00	8.52
Temperature (ºC)					
17.0	19,150	2.78	1.51	0.00	8.52
17.5	29,072	2.50	1.42	0.00	8.34
18.0	1,954	2.70	1.44	0.00	7.35
18.5	1,138	2.83	1.50	0.00	7.91
19.0	14,350	2.80	1.51	0.00	8.42
19.5	1,542	2.93	1.48	0.00	8.07

n, number of bulls included in the analysis; SD, standard deviation; Min., minimum yield; Max., maximum yield.

**Table 2:** Parameters estimation at different temperature gradients for somatic cell score of Holstein cattle in southern Brazil

Temperature (ºC)	σ^2^_p_	σ^2^_e_	σ^2^_a_	h^2^	SE
17.0	1.81	1.45	0.36	0.20	0.146
17.5	1.64	1.32	0.32	0.19	0.019
18.0	1.67	1.37	0.30	0.18	0.008
18.5	1.78	1.48	0.31	0.17	0.019
19.0	1.75	1.41	0.34	0.19	0.017
19.5	1.84	1.43	0.40	0.23	0.033

σ^2^_p_, phenotypic variance; σ^2^_e_, environmental variance; σ^2^_a_, genetic variance; h^2^, heritability; SE, standard error.
